# Medical students’ thought process while solving problems in 3 different types of clinical assessments in Korea: clinical performance examination, multimedia case-based assessment, and modified essay question

**DOI:** 10.3352/jeehp.2019.16.10

**Published:** 2019-05-09

**Authors:** Sejin Kim, Ikseon Choi, Bo Young Yoon, Min Jeong Kwon, Seok-jin Choi, Sang Hyun Kim, Jong-Tae Lee, Byoung Doo Rhee

**Affiliations:** 1 Research and Innovation in Learning Lab, College of Education, The University of Georgia, Athens, GA, USA; 2 Department of Internal Medicine, Inje University College of Medicine, Busan, Korea; 3 Department of Radiology, Inje University College of Medicine, Busan, Korea; 4 Department of Preventive Medicine, Inje University College of Medicine, Busan, Korea; Hallym University, Korea

**Keywords:** Clinical performance examination, Modified essay question, Case-based assessment, Clinical assessment, Assessment validity, Republic of Korea

## Abstract

**Purpose:**

This study aimed to explore students’ cognitive patterns while solving clinical problems in 3 different types of assessments—clinical performance examination (CPX), multimedia case-based assessment (CBA), and modified essay question (MEQ)—and thereby to understand how different types of assessments stimulate different patterns of thinking.

**Methods:**

A total of 6 test-performance cases from 2 fourth-year medical students were used in this cross-case study. Data were collected through one-on-one interviews using a stimulated recall protocol where students were shown videos of themselves taking each assessment and asked to elaborate on what they were thinking. The unit of analysis was the smallest phrases or sentences in the participants’ narratives that represented a meaningful cognitive occurrence. The narrative data were reorganized chronologically and then analyzed according to the hypothetico-deductive reasoning framework for clinical reasoning.

**Results:**

Both participants demonstrated similar proportional frequencies of clinical reasoning patterns on the same clinical assessments. The results also revealed that the three different assessment types may stimulate different patterns of clinical reasoning. For example, the CPX strongly promoted the participants’ reasoning related to inquiry strategy, while the MEQ strongly promoted hypothesis generation. Similarly, data analysis and synthesis by the participants were more strongly stimulated by the CBA than by the other assessment types.

**Conclusion:**

This study found that different assessment designs stimulated different patterns of thinking during problem-solving. This finding can contribute to the search for ways to improve current clinical assessments. Importantly, the research method used in this study can be utilized as an alternative way to examine the validity of clinical assessments.

## Introduction

How do medical students actually think while they are solving clinical problems in testing situations? Understanding the kinds of thinking students are actually engaged in while taking tests is essential for validating and improving clinical assessments. While most research in this area has focused on the results of clinical assessments [1,2], little attention has been given to exploring students’ thought process during tests. Thus, the purpose of this study was to explore medical students’ cognitive patterns while solving clinical diagnostic problems in different types of clinical assessments and to compare those cognitive patterns across the different assessments.

The objective structured clinical examination and clinical performance examination (CPX) have been widely used in clinical assessments as standardized ways to assess components of clinical competence beyond general knowledge, such as clinical performance and reasoning [1-4]. The modified essay question (MEQ) is also frequently used as a paper-based method to assess clinical reasoning [5,6]. In this study, in order to explore and compare medical students’ cognitive patterns when completing different types of clinical assessments, we first chose 2 representative types of clinical assessments commonly used in medical education: the CPX as a clinical performance test and the MEQ as a paper-based clinical reasoning test. In addition, we included an emerging alternative assessment, a multimedia case-based assessment (CBA) that was designed and developed by Choi et al. [7]. Therefore, this study was conducted to answer the following research questions: (1) Research question 1: What are medical students’ cognitive patterns of clinical diagnostic problem-solving in 3 different types of clinical assessments: the CPX, CBA, and MEQ? (2) Research question 2: How do medical students think differently to solve clinical diagnostic problems in 3 different types of clinical assessments: the CPX, CBA, and MEQ?

## Methods

### Ethics statement

This study was approved by the Institutional Review Board of the University of Georgia (STUDY00001057). Written informed consent was obtained by the researchers.

### Research design and intervention design

**Fig. 1.**
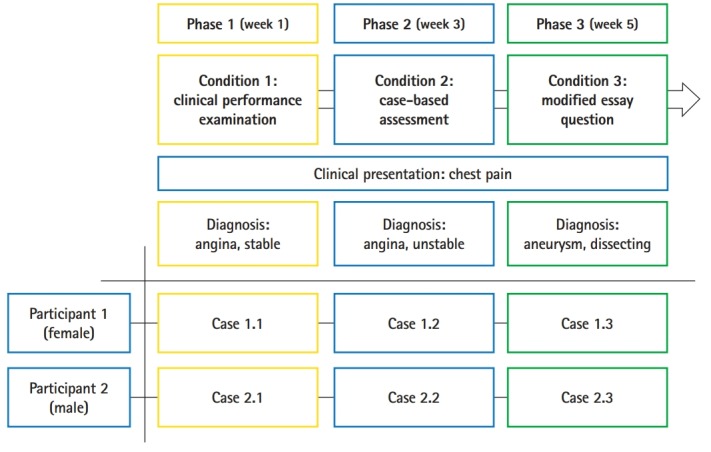
Research design, intervention design, and procedure: 3 different types of conditions.

A cross-case analysis was employed with 2 research participants and 3 different types of assessments in order to compare the differences in their cognitive patterns. The participants completed each type of assessment in the order of the condition numbers: condition 1 for the CPX, condition 2 for the CBA, and condition 3 for the MEQ (Fig. 1). The interval between each test was 2 weeks. Therefore, a total of 6 test-performance cases were collected and analyzed. The key features of the CPX, CBA, and MEQ used in this study are listed below:

#### CPX

A 10-minute standardized patient-based clinical examination where a student interacts with a person who was recruited and trained to act as a real patient by simulating specific symptoms. The student’s clinical performance, including history-taking, physical examination, and diagnostic decisions, are assessed by an evaluation rubric.

#### CBA

A video-based examination where a clinical case divided into 4 segments is provided and followed by a set of questions for each segment to assess a student’s clinical reasoning process and decision-making. The questions are related to cue identification, hypothesis generation, inquiry strategy, and data analysis for diagnosis. The test is delivered through an Internet browser on a computer, and the student cannot go back to the previous segment to change the answers. Each segment has a time limit (first segment, 6.5 minutes; second segment, 12 minutes; third segment, 12 minutes; and fourth segment, 17.5 minutes).

#### MEQ

A paper-based clinical examination where a clinical case is provided on paper along with a sequence of questions to assess a student’s clinical reasoning and decision-making. The test items include questions regarding cue identification, the diagnostic algorithm, hypothesis generation, and diagnosis. The MEQ in this study was composed of 2 segments, providing a sequence of scenarios on different pages with different stages of a clinical problem. Each segment had a 10-minute time limit for the student to solve all questions.

A clinical presentation of chest pain was selected as the subject matter for all 3 assessments in the study. Chest pain is an essential clinical presentation regularly used in medical school curricula [8], which helped to control for participants’ lack of knowledge as a factor in problem-solving. This choice of subject matter also favored participants’ use of reasoning behaviors instead of hunting or guessing for the right answers. To prevent learning effects from taking 3 similar tests consecutively, the final diagnosis for each assessment was different—angina, stable; angina, unstable; and aneurysm, dissecting for the CPX, CBA, and MEQ, respectively—although chest pain was the chief complaint in each assessment.

### Participants

Due to the high level of complexity and sensitivity in data collection and analysis, we aimed to recruit 2 fourth-year students from a medical school in Busan, Korea. In order to minimize unintended influence on participants’ thinking processes during the assessments from a lack of prior knowledge and CPX skills, purposeful sampling was employed. We first targeted fourth-year medical students; among a cohort of 109 fourth-year students, 17 who had experienced academic failure in previous semesters and 2 foreign students whose first language was not Korean were excluded. Next, we targeted the top 25% of the remining 90 students based on academic achievement, using their grade point average from the previous 3 years. The participants had completed the first semester of the fourth year by the time they participated in this study. This reduced the likelihood that participants simply guessed during problem-solving due to a lack of prior knowledge. Furthermore, in order to eliminate outliers in CPX performance, only those who had above-average previous CPX scores were targeted for recruitment. Finally, 1 female student and 1 male student were recruited to ensure gender balance in the study.

### Data collection

This study utilized a video-based stimulated recall protocol for interviews that included 2 steps of data collection for each case. In step 1, the participants’ performances in each case was video-recorded. Then, each captured video was divided into 20 segments. For example, each segment of a 10-minute CPX video captured 30 seconds of the participant’s performance. Likewise, the participants’ CBA and MEQ performance videos were also divided into 20 segments, but the actual duration of each segment varied according to the total time of each performance. In step 2, each segment of the video was played back to the participants, and while watching their own performance on video, they were asked to recall cognitive occurrences by answering questions posed by the interviewer [9]. A list of retrospective interview questions used to elicit cognitive occurrences from the participants is provided in Table 1.

### Data analysis

The interview data from the 6 cases (2 participants each with 3 test conditions) were transcribed and analyzed based on the hypothetico-deductive reasoning (HDR) model to identify the participants’ cognitive patterns of clinical diagnostic problem-solving. The HDR is one of the most suitable models for medical students to apply and practice their clinical reasoning to make a diagnosis [10,11]. The unit of analysis in this study was the smallest phrases or sentences from the participants’ narratives that represented a meaningful cognitive occurrence The transcribed narrative data were chunked by the unit of analysis as cognitive occurrences, guided by a naturalistic decision-making model (NDM) [9,12,13]. A sample data table with the unit of analysis (cognitive occurrence), NDM cognitive element category, and content of cognition is provided in Table 2.

For further data analysis, each unit of the narrative data was chronologically reorganized in order to reconstruct the participants’ cognitive processes according to the order of the actual events that occurred during their performances. The reorganized data were then coded according to the HDR model, and any uncoded data were categorized as other cognitive occurrences. The category of other cognitive occurrences included cognitive behaviors that may not be authentic in real-world settings and may not necessarily occur when doctors encounter patients, but instead occur only in certain testing contexts. Because the other cognitive occurrences were not the main focus of this study, they were not subdivided or analyzed further. To obtain an overall picture of students’ cognitive patterns, however, the related statistics for other cognitive occurrences were still reported. A sample of a data analysis table with the reconstructed cognitive occurrences in chronological order, HDR coding indications, and other cognitive occurrence themes is presented in Table 3.

### Inter-rater reliability

Inter-rater reliability was assessed in order to ensure the accuracy of the findings and the consistency of the analysis procedures. Two raters (the first author, an education expert, and the fourth author, a clinical expert) completed training sessions with the second author (an education expert). Then, the 2 raters coded the data independently according to the HDR model. Their independent coding results were compared, and any disagreements in the coding results between the 2 raters were identified and negotiated. The inter-rater reliability results for the initial and final coding are provided in Table 4.

## Results

### Identification of clinical reasoning patterns and other cognitive occurrences in each condition (research question 1)

In order to answer the first research question, the 2 participants’ cognitive patterns were compared. The proportional data (%) of each type of cognitive occurrences in each case, instead of the actual frequencies, were used in this study because each participant’s cognitive processes were based on their narrative data, the lengths of which were not equal. As shown in Table 5, 79.7% and 69.4% of the cognitive occurrences in participant 1 (female) and 2 (male), respectively, were identified as clinical reasoning during the CPX condition, while 20.3% and 30.6% of the cognitive occurrences, respectively, were found to be other cognitive occurrences. Likewise, 61.4% and 86.6% of the cognitive occurrences in participant 1 and 2, respectively, were clinical reasoning during the CBA condition, and 38.6% and 13.4% of the cognitive occurrences, respectively, were other cognitive occurrences. During the MEQ condition, 63.7% and 67.0% of the cognitive occurrences in participant 1 and 2, respectively, were identified as clinical reasoning, while 36.3% and 33.0% of the cognitive occurrences, respectively, were found to be other cognitive occurrences. As indicated earlier, the other cognitive occurrences consisted of various kinds of inauthentic thinking. The further analysis focused primarily on the cognitive occurrences classified as clinical reasoning.

**Fig. 2.**
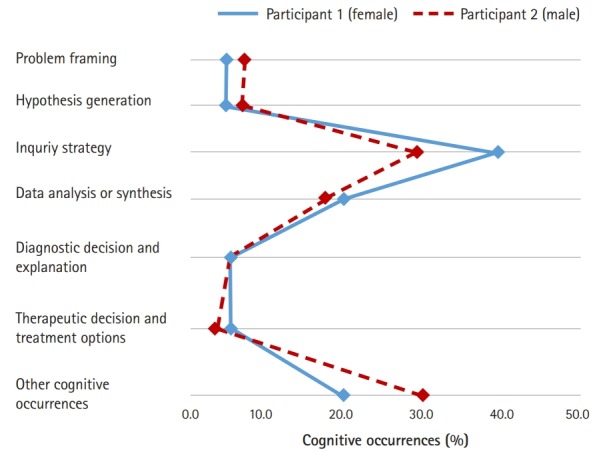
The 2 participants’ proportional patterns of cognitive occurrences in condition 1 (clinical performance examination).

In order to explore the similarities of the proportional patterns of clinical reasoning between the 2 participants in each condition, line graphs were used to represent the results of Table 5 graphically. As shown in Fig. 2, a similar proportional pattern of clinical reasoning between both participants was evident in the CPX condition. In general, more clinical reasoning was observed in participant 1 (female) than in participant 2 (male). The inquiry strategy phase (39.8% for participant 1 and 28.9% for participant 2) was the most frequent clinical reasoning process for both participants, and the data analysis or synthesis phase (20.3% for participant 1 and 17.9% for participant 2) was the next most frequent.

**Fig. 3.**
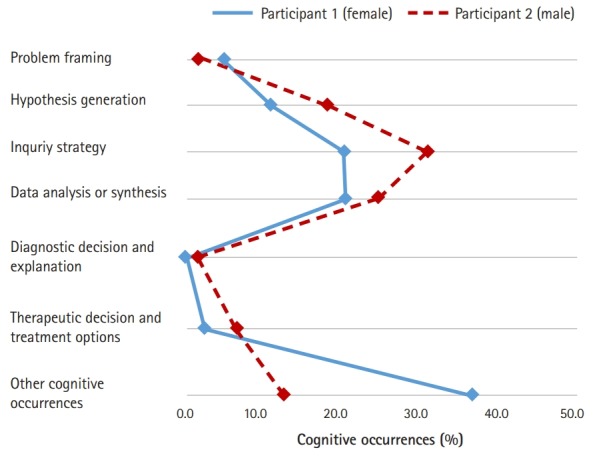
The 2 participants’ proportional patterns of cognitive occurrences in condition 2 (multimedia case-based assessment).

Likewise, similar proportional patterns of clinical reasoning were observed between the 2 participants in the CBA condition (Fig. 3). The inquiry strategy phase (21.0% for participant 1 and 32.3% for participant 2) and the data analysis or synthesis phase (21.5% for participant 1 and 25.4% for participant 2) were the 2 most frequent types of cognitive occurrences.

**Fig. 4.**
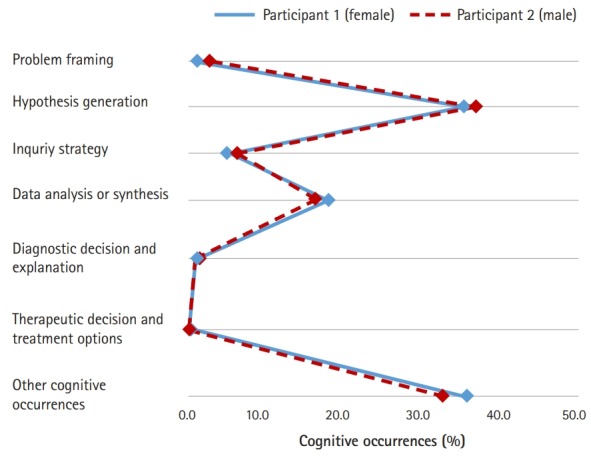
The 2 participants’ proportional patterns of cognitive occurrences in condition 3 (modified essay question).

Similarly, both participants’ proportional patterns of clinical reasoning were almost identical in the MEQ condition, as presented in Fig. 4. The hypothesis generation phase (36.8% for participant 1 and 38.0% for participant 2) was the most frequent clinical reasoning process for both participants, and the data analysis or synthesis phase (18.1% for participant 1 and 16.8% for participant 2) was the next most frequent.

### Differences in cognitive patterns among the 3 different conditions (research question 2)

In order to explore the differences among the participants’ cognitive patterns facilitated by each condition, the average percentages of the types of cognitive occurrences from both participants across all conditions were compared. The average percentages for clinical reasoning were 74.6% (CPX), 74.0% (CBA), and 65.4% (MEQ), whereas the average percentages for other cognitive occurrences across all conditions were 25.4% (CPX), 26.0% (CBA), and 34.6% (MEQ) (see Table 5 for the detailed average percentages of each HDR process and other cognitive occurrences).

**Fig. 5.**
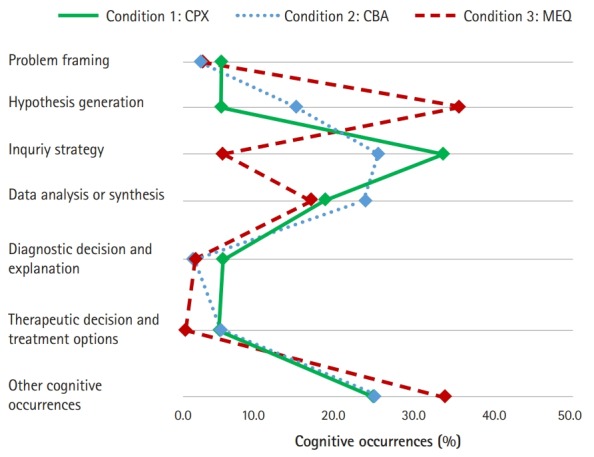
The average patterns of the 2 participants’ proportional cognitive occurrences in the 3 conditions (CPX, CBA, and MEQ). CPX, clinical performance examination; CBA, multimedia case-based assessment; MEQ, modified essay question.

The average values of the 2 participants’ proportional patterns of clinical reasoning in the 3 different assessment conditions are demonstrated in Fig. 5. In the MEQ condition, hypothesis generation was observed more frequently than any other condition, while more inquiry strategies were observed in the CPX and CBA conditions than in the MEQ condition. Clinical reasoning related to data analysis and synthesis was observed in all 3 conditions at a similar level. Clinical reasoning related to problem framing, diagnostic decision and explanation and therapeutic decision and treatment options was observed infrequently in all 3 conditions.

## Discussion

There are 2 key findings of this study. First, for each condition, the cognitive patterns of the 2 participants were similar to each other (Figs. 2–4). This implies that each assessment type may stimulate thinking in a consistent manner across multiple test-takers. The first finding is the precondition for the second finding, which is that the three different assessment types stimulated different aspects of clinical reasoning. As shown in Fig. 5, the CPX promoted reasoning involving inquiry strategy, but it rarely promoted hypothesis generation. By design, one of the key features of the CPX is to provide a unique situation where test-takers freely interact with standardized patients by asking questions during the diagnostic process [1,3]. Thus test-takers mostly take advantage of being engaged in dynamic inquiry activities during the CPX, although they may not have enough time to think carefully in order to formulate hypotheses. In contrast, the MEQ strongly promoted hypothesis generation, but not inquiry strategy. The MEQ includes an item that requests test-takers to articulate hypotheses for a given situation, which directly stimulates their engagement in reasoning related to hypothesis generation. Due to the static nature of the paper-based mode, the MEQ does not have a dynamic mechanism through which test-takers could generate a list of questions to obtain corresponding patient information. Interestingly, the CBA strongly promoted inquiry strategy at a level close to that of the CPX, while the CBA promoted hypothesis generation at a level higher than the CPX, but lower than the MEQ. The key features of the CBA include a series of consecutive video clips divided by critical decision points where test-takers are given clips sequentially and asked to reason at that given particular moment. With the multimedia feature delivering richer situational information in an interactive mode, the CBA might be able to provide test-takers opportunities to practice both inquiry strategies and hypothesis generation, which were not promoted simultaneously by the other assessment types.

Four limitations of this study can be noted. First, generalization of the findings of the study is limited by the small sample size of participants and the inclusion of a single clinical presentation. Secondly, the collected data were based on the participants’ recalled narratives, which might lopsidedly reflect positive outcomes due to their status as high-performing medical students; furthermore, the students might have missed some important cognitive occurrences when they were watching their performance videos. Thirdly, a specific test item for the therapeutic decision and treatment options was not included in the MEQ used in this study; hence, this particular cognitive process was not detected in the MEQ for either participant. Lastly, test-retest bias may have affected our findings, as 3 different tests with similar content were consecutively administered to each participant.

In conclusion, we argue that different assessment designs stimulate different patterns of thinking; thus, they may measure different aspects of cognitive performance. Although each assessment was designed with the same content (e.g., chest pain) and the same goal (e.g., assessing clinical reasoning), the combination of different delivery modes and the different ways that each test item is designed may cause variation in students’ actual thought processes while taking the test. It is essential to strive to understand which aspects of cognitive performance are actually measured in different assessments in order to improve such assessments. Accordingly, we recommend that researchers consider the research method employed in this study, capturing and analyzing data on cognitive occurrences, as an alternative method for examining the validity of assessment instruments, in particular for construct validity. For example, our method allowed us to discover discrepancies between participants’ observed performance and their actual thought process for the CPX. While the participants were using a stethoscope during the physical examination part, their attention was not directed towards the task at hand; instead, they focused on matters such as next steps in the physical examination, and information-retrieving for the patient education and counseling part. These inattentive actions, coded as other cognitive occurrences in this study, may not be detected through expert observation that uses a performance checklist in the current design of the CPX. Thus, through the method utilized in this study, it is possible to check whether a certain assessment instrument is valid for assessing the intended aspects of cognitive performance.

## Figures and Tables

**Table 1.** Retrospective interview questions for video-stimulated recall of each condition

Type	Interview question	Timing
General questions	What did you see here, if you can recall the moment in this (time) video clip?	At each segment
	What did you hear here, if you can recall the moment in this (time) video clip?	
	What did you read here, if you can recall the moment in this (time) video clip?	
	What did you feel here, if you can recall the moment in this (time) video clip?	
	What did you do or think at that moment?	
	Why did you think this way?	
	Why did you make that decision at that moment?	
	Why did you do it this way?	
	Did you plan ahead for the next step? What did you expect would happen after this?	
Additional questions	Did you think about the meaning of what you saw, heard, read, and felt? What was that meaning?	If needed
	Did you make a decision at that moment? What was it?	
	Can you explain why you did this?	
	What was the most challenging experience while you solved the problem?	

**Table 2.** A sample of a data analysis table with occurrence, NDM model classification, and decision content

Original transcript	Original no.	Unit of analysis (cognitive occurrence)	NDM cognitive element category	Content of cognition
First, the reason why I asked the patient about more details of her chest pain was to get more information from open-ended questions. But because she didn’t give me much information, I asked closed questions one-by-one. Initially (I asked about) location, duration (how long each painful episode is), timeframe (when the pain began) Because these can be significant clues to the diagnosis, I asked these questions first. So, I asked: “Can you point to where it hurts?” instead of asking “where does it hurt?” in order to get a more accurate location of the pain. At that time the patient pointed to the middle of her chest and said: “It hurts here.” After I found the location of the chest pain, I was thinking of what I needed to ask next for the differential diagnosis. There are many possibilities for pain in the center of the chest; for example, heart disease… So I thought I needed to keep my (diagnostic) options open. For example, although she pointed at the middle of her chest, I was thinking, was it really the front part of her chest or could it be the back part? Also, it may not even be heart disease. So, I thought I had to keep my options open.	33	First, the reason why I asked the patient about more details of her chest pain	Rationale	Reason of the pain
	34	was to get more information from open-ended questions.	Rationale	Open question
	35	But because she didn’t give me much information,	Information	Information
	36	I asked closed questions one-by-one.	Rationale	Close question
	37	Initially (I asked about) location, duration (how long each painful episode is), timeframe (when the pain began)	Cue identification	Location, duration, & timeframe
	38	Because these can be significant clues to the diagnosis,	Rationale	Diagnosis
	39	I asked these questions first.	Cue identification	Location, duration, & timeframe
	40	So, I asked: “Can you point to where it hurts?”	Cue identification	Location
	41	instead of asking “where does it hurt?” in order to get a more accurate location of the pain.	Rationale	Location
	42	in order to get a more accurate location of the pain.	Rationale	Location
	43	At that time the patient pointed to the middle of her chest and said: “It hurts here.”	Cue interpretation	Location
	44	After I found the location of the chest pain,	Information	Location
	45	I was thinking of what I needed to ask next for the differential diagnosis.	Goal setting	Diagnosis
	46	There are many possibilities for pain in the center of the chest; for example, heart disease…	Appraisal	Hypothesis
	47	So I thought I needed to keep my (diagnostic) options open.	Decision-making	Hypothesis
	48	For example, although she pointed at the middle of her chest, I was thinking was it really the front part of her chest or could it be the back part?	Rationale	Location
	49	Also, it may not even be heart disease.	Rationale	Hypothesis
	50	So, I thought I had to keep my options open.	Decision-making	Hypothesis

NDM, naturalistic decision-making.

**Table 3.** A sample of a data analysis table with HDR coding and other cognitive occurrences

Chronically rearranged no.	Original no.	Chronically reconstructed cognitive occurrence	NDM cognitive element category	Content of cognition	HDR coding	Other cognitive occurrences
252	289	But this time I omitted tapping and touching,	Decision-making	Physical examination	Inquiry strategy	
253	290	Because I thought the possibility of it not being respiratory disease was higher than other (conditions)…	Rationale	Hypothesis	Data analysis or synthesis	
254	293	For the respiratory symptoms… I had already asked quite a lot of questions to carry out the differential diagnosis for the respiratory system.	Cue identification	History-taking	Inquiry strategy	
255	294	So, I thought I was finished with the respiratory system,	Anticipation	Diagnosis	Data analysis or synthesis	
256	291	A lot of time had already passed, and I didn’t think I needed to do it.	Appraisal	Physical examination	Inquiry strategy	
257	292	So, I skipped those two steps: tapping and touching	Goal setting	Physical examination		Off-protocol behavior
258	295	And I didn’t do tapping and touching because of the time limit.	Self-reflection	Time pressure		Off-protocol behavior
259	296	But I was not sure about the criteria of the checklist (gradebook) so I thought if I did not do it… the mission was to do a physical examination for chest pain, but if I did not do tapping and touching… what if I can’t get a score for this?	Self-reflection	Grading criteria		Point-seeking/hunting
260	297	Then I had just wasted my time…	Self-reflection	Grading criteria		Off-protocol behavior
261	298	First, I thought I could do a differential diagnosis with only the stethoscope for the heart and the lung for the disease I was thinking about.	Self-reflection	Physical examination	Physical examination	
262	299	Then the next step was asking the patient, when I pressed here, if the patient felt pain,	Plans	Physical examination	Physical examination	
263	300	(To see) if there were any pressure points.	Plans	Physical examination	Physical examination	

HDR, hypothetico-deductive reasoning; NDM, naturalistic decision-making.

**Table 4.** Inter-rater reliability results of the 2 raters’ coding

Condition	Case	Cognitive process	Total no. of codes	Initial agreement (%)	Second agreement (%)
Condition 1: CPX	Case 1.1	HDR process	98	74.8	100.0
		Other cognitive occurrences	25	67.9	100.0
	Case 1.2	HDR process	209	78.6	100.0
		Other cognitive occurrences	92	74.8	100.0
Condition 2: CBA	Case 2.1	HDR process	143	81.0	100.0
		Other cognitive occurrences	90	93.7	100.0
	Case 2.2	HDR process	174	86.1	100.0
		Other cognitive occurrences	27	87.1	100.0
Condition 3: MEQ	Case 3.1	HDR process	109	82.9	100.0
		Other cognitive occurrences	62	86.3	100.0
	Case 3.2	HDR process	120	97.5	100.0
		Other cognitive occurrences	59	97.6	100.0

CPX, clinical performance examination; HDR, hypothetico-deductive reasoning; CBA, multimedia case-based assessment; MEQ, modified essay question.

**Table 5.** Clinical reasoning and other cognitive occurrences in the 3 conditions

Variable	Category	Condition 1: CPX	Condition 2: CBA	Condition 3: MEQ
Clinical reasoning	Participant 1 (female)	79.7 (98)	61.4 (143)	63.7 (109)
	Participant 2 (male)	69.4 (209)	86.6 (174)	67.0 (120)
	Average	74.6	74.0	65.4
Problem framing	Participant 1 (female)	4.1 (5)	4.3 (10)	1.8 (3)
	Participant 2 (male)	7.0 (21)	1.5 (3)	3.9 (7)
	Average	5.5	2.9	2.8
Hypothesis generation	Participant 1 (female)	4.1 (5)	11.6 (27)	36.8 (63)
	Participant 2 (male)	6.6 (20)	18.4 (37)	38.0 (68)
	Average	5.4	15.0	37.4
Inquiry strategy	Participant 1 (female)	39.8 (49)	21.0 (49)	5.9 (10)
	Participant 2 (male)	28.9 (87)	32.3 (65)	6.7 (12)
	Average	34.4	26.7	6.3
Data analysis or synthesis	Participant 1 (female)	20.3 (25)	21.5 (50)	18.1 (31)
	Participant 2 (male)	17.9 (54)	25.4 (51)	16.8 (30)
	Average	19.1	23.4	17.5
Diagnostic decision and explanation	Participant 1 (female)	5.7 (7)	0	1.2 (2)
	Participant 2 (male)	5.3 (16)	2.0 (4)	1.7 (3)
	Average	5.5	1.0	1.4
Therapeutic decision and treatment options	Participant 1 (female)	5.7 (7)	3.0 (7)	0
	Participant 2 (male)	3.7 (11)	7.0 (14)	0
	Average	4.7	5.0	0
Other cognitive occurrences	Participant 1 (female)	20.3 (25)	38.6 (90)	36.3 (62)
	Participant 2 (male)	30.6 (92)	13.4 (27)	33.0 (59)
	Average	25.4	26.0	34.6
Total	Participant 1 (female)	100.0 (123)	100.0 (233)	100.0 (171)
	Participant 2 (male)	100.0 (301)	100.0 (201)	100.0 (179)

Values are presented as % (frequency) or %. CPX, clinical performance examination; CBA, multimedia case-based assessment; MEQ, modified essay question.

## Supplementary materials

Supplement 1. Audio recording of the abstract.
